# Hazards of tube thoracostomy in patients on a ventilator

**DOI:** 10.1186/1749-8090-6-39

**Published:** 2011-03-29

**Authors:** Kasra Shaikhrezai, Vipin Zamvar

**Affiliations:** 1 Department of Cardiothoracic Surgery, Royal Infirmary of Edinburgh, Edinburgh, UK

## Abstract

A patient with post-pneumonia empyema complicated by type-2 respiratory failure required mechanical ventilation as part of his therapy. A pneumothorax was noted on his chest radiograph. This was treated with an intercostal chest drain (ICD). Unfortunately, he was still hypoxic, his subcutaneous emphysema was worsening and the ICD was bubbling. A computed tomography (CT) scan of chest demonstrated that the ICD has penetrated the right upper lobe parenchyma. A new ICD was inserted and the previous one was removed. Although both hypoxia and subcutaneous emphysema improved, the patient chronically remained on mechanical ventilation.

## Background

Tube thoracostomy is a common procedure to drain fluids and/or air from the pleural space via an ICD. The British Thoracic Society (BTS) has published a guideline [1] for ICD insertion which in many institutions has been deployed as a standard approach to tube thoracostomy in both practice and training programs. Recently there is an increasing concern regarding the training of doctors with regard to precise and methodological ICD insertion [2,3]. Harris et al [4] conducted a national survey among chest physicians in the UK recording their experiences regarding complications and serious harms following ICD insertion. The study revealed 67% of NHS trusts have experienced major complications of ICD insertion.

## Case presentation

**Figure 1:**
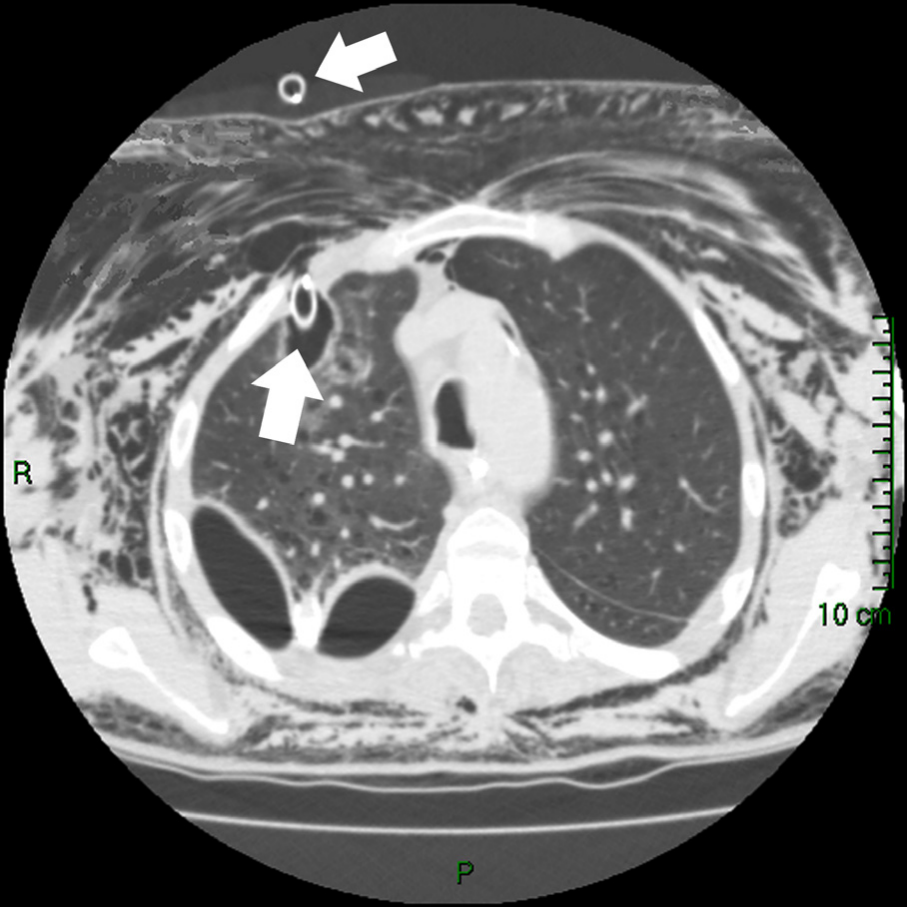
**ICD (arrows) penetrating the lung parenchyma**.

A 51-year-old man with history of chronic obstructive pulmonary disease (COPD) and cigarette smoking presented with a shortness of breath, chronic pneumonia and empyema involving the right side of his chest. Soon after admission his condition deteriorated developing type-2 respiratory failure necessitating intubation and commencement of mechanical ventilation. Patient required positive end-expiratory pressure (PEEP) of 10 mmHg and 80% fraction of inspired oxygen (FiO2) to maintain the oxygen saturation of 91% with PCO2 (partial pressure of carbon dioxide) and PO2 (partial pressure of oxygen) of 7.1 and 8.2 kPa respectively. Following central line insertion a pneumothorax was noted on his chest radiograph. Under aseptic technique and blunt dissection a large bore ICD was inserted anterolaterally into the right chest preceded by the introduction of index finger and sweeping manoeuvre explained by the BTS guidelines [1]. It is imperative to appreciate that a diseased hyperventilated lung with a high PEEP is very prone to perforation by any instruments penetrating the chest wall and pleura. Shortly after tube thoracostomy the patient started to develop a large subcutaneous emphysema originating in the right moving towards the left side of the chest wall. Unfortunately his hypoxic state became worse requiring augmentation of mechanical ventilation. In the interim ICD was bubbling constantly. A CT scan of chest demonstrated that the ICD has penetrated the right upper lobe parenchyma (Figure 1). As a result patient was urgently transferred to our institute for further management.

A new ICD was inserted with the same technique whilst the ventilator was briefly disconnected. When it was proved that the new ICD is in the appropriate position with a characteristic swing of column of water, the previous ICD was removed.

**Figure 2:**
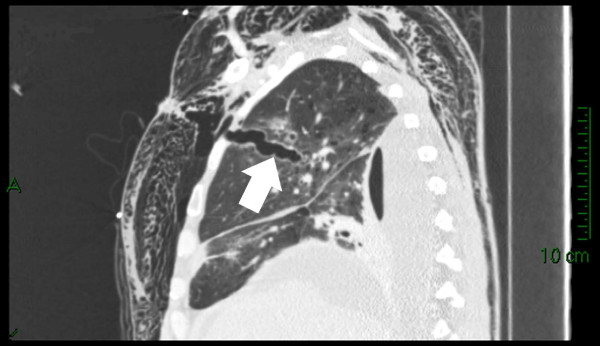
**Right upper lobe laceration (arrow) containing gas communicating with the anterior chest wall (post ICD removal)**.

Subsequent chest CT scan revealed the right upper lobe laceration containing gas communicating with the anterior chest wall. This was accompanied by massive subcutaneous emphysema (Figure 2).

Although following the new ICD both hypoxia and subcutaneous emphysema improved the patient was chronically remained on ventilation.

## Conclusion

Previously the risks of ICD insertion in patients on mechanical ventilation has been described [5] however we presented the above case due to frequent referral of patients on mechanical ventilation to us with harmful complications of tube thoracostomy. Prior to ICD insertion in a patient on mechanical ventilation, the PEEP must be turned off and the ventilator must be disconnected briefly during the introduction of the ICD. In ICD insertion deploying Seldinger technique the same steps need to be taken for introducing the guide wire as well as the chest tube. Any ICD breaching the lung parenchyma should be removed after insertion of another ICD in the pleural space.

We believe the BTS guidelines [1] require a new revision with the view to including the mechanical ventilation as a hazardous clinical setting in "pre-drainage risk assessment" section. Furthermore ICD insertion needs to be explained separately in self- and mechanical-ventilating patients along with considering the clinical settings as well as the specialty demands.

For instance efficient drainage of left-sided pleural effusion in a post-CABG (coronary artery bypass graft surgery) patient requires a tube thoracostomy below the triangle of safety; or fine bore ICD insertion under Seldinger technique for the treatment of pneumothorax is a well established procedure deployed by respiratory physicians while in thoracic surgery a large bore ICD with conventional insertion technique is favourable.

The royal college of surgeons has introduced S-DOPS (direct observation of procedural skills in surgery) via intercollegiate surgical curriculum programme (ISCP) [6]. We recommend a unified usage of surgical DOPS in all specialties to sign off junior doctors' competency in tube thoracostomy in self- and mechanical-ventilating patients.

## Consent

Written informed consent was obtained from the patient for publication of this case report and accompanying images. A copy of the written consent is available for review by the Editor-in-Chief of this journal.

## Competing interests

The authors declare that they have no competing interests.

## Authors' contributions

KS performed the procedure; VZ admitted the patient under his care, instructed and supervised the procedure. All authors read and approved the final manuscript.
